# Identification and Characterization of the Growth-Regulating Factors-Interacting Factors in Cotton

**DOI:** 10.3389/fgene.2022.851343

**Published:** 2022-03-14

**Authors:** Daowu Hu, Yuting Ge, Yinhua Jia, Shoupu He, Xiaoli Geng, Liru Wang, Zhaoe Pan, Zubair Iqbal, Tahir Mahmood, Hongge Li, Baojun Chen, Xiaoyang Wang, Baoyin Pang, Xiongming Du

**Affiliations:** Institute of Cotton Research of Chinese Academy of Agricultural Science/State Key Laboratory of Cotton Biology, Anyang, China

**Keywords:** cotton, GIF, lateral root, waterlogging, VIGS

## Abstract

Growth-regulating factors-interacting factors (GIFs) are a type of transcription co-activators in plants, playing crucial roles in plants’ growth, development, and stress adaptation. Here, a total of 35 *GIF* genes were identified and clustered into two groups by phylogenetic analysis in four cotton genus. The gene structure and conserved domain analysis proved the conservative characteristics of *GIF* genes in cotton. The function of *GIF* genes was evaluated in two cotton accessions, Ji A-1-7 (33xi) and King, which have larger and smaller lateral root numbers, respectively. The results showed that the expression of *GhGIF4* in Ji A-1-7 (33xi) was higher than that in King. The enzyme activity and microstructure assay showed a higher POD activity, lower MDA content, and more giant cells of the lateral root emergence part phenotype in Ji A-1-7 (33xi) than in King. A mild waterlogging assay showed the *GIF* genes were down-regulated in the waterlogged seedling. Further confirmation of the suppression of *GhGIF4* in cotton plants further confirmed that *GhGIF4* could reduce the lateral root numbers in cotton. This study could provide a basis for future studies of the role of *GIF* genes in upland cotton.

## 1 Introduction

Growth-regulating factors-interacting factors (GIFs) are a class of transcription co-activators of growth-regulating factors (GRFs). *GIFs* usually interact with GRFs to form a plant-specific transcriptional complex (Kim and Tsukaya, 2015). The *AtGIF* gene family was first described by Kim in *Arabidopsis* in 2004 (Kim and Kende, 2004). It was shown that the *GIF1* gene could act as a functional homolog of the human *SYT* transcription coactivator. Subsequently, *GIF* genes were associated with plant growth and development (Horiguchi et al., 2005; Vercruyssen et al., 2014).

Previous research showed that *GIF1* positively regulated cell proliferation in lateral organs like leaves and petals of *Arabidopsis*. A narrower and smaller leaf phenotype (than control), which was caused by reduced cell numbers along the leaf-width axis, was observed in the *gif1* mutant and transgenic plants, indicating that *AtGIF1* may function as a transcriptional co-activator that is involved in regulating the growth and shape of plant leaves and petals (Kim and Kende, 2004). *AtGIF1* was also called *AN3* (*ANGUSTIFOLIA3*) in *Arabidopsis*. Another study confirmed the above result by revealing that *an3* and *grf5* mutations exhibited a narrow-leaf phenotype due to reduced of cell numbers, while overexpression of *AN3/GRF5* increased leaf size with standard shape (Horiguchi et al., 2005). *AN3* could interact with the *SWI/SNF* chromatin remodeling complexes to regulate leaf development during the transition period from cell proliferation to cell differentiation (Vercruyssen et al., 2014). It was also found *GIF* regulates the formation of the pistil in *Arabidopsis* and single *gif* mutant lines showed a similar phenotype to control, but the *gif* triple mutant *gif1/gif2/gif3* exhibited an abnormal pistil (Liang et al., 2014). A recent research (Ercoli et al., 2018) revealed that the *GIFs* are necessary to maintain the precise expression patterns of key developmental factors, and the *GIF* transcriptional coregulators could control QC (the quiescent center) organization and the size of the meristem in *Arabidopsis* (Ercoli et al., 2018). *GIF2* and *GIF3* are two additional proteins involved in cell proliferation and lateral organ growth development (Lee et al., 2009). The *gif2* and *gif3* mutated plants accommodate a smaller lateral organ than wild type plant species, caused by the synergistic reduction in cell numbers (Lee et al., 2009). Recent data shows that GRFs alone or in chimeras with *GIFs* could dramatically boost regeneration from tissue cultures in a broad range of plant species which is significant for plant transformation and gene editing (Debernardi et al., 2020; Kong et al., 2020; Luo and Palmgren, 2020).

The role of *GIFs* has also been in the regulation of plant growth and the development of crops. *OsGIF1/2/3* with *OsGRF4* controls the grain size and weight in rice, resulting from increased and heavier grain yield (Duan et al., 2015). *GIF1* was also to be involved in the regulation of shoot architecture and meristem determinacy in maize (Zhang et al., 2018). Four *SlGIF* genes were identified to interact with various SlGRF proteins to affect the development of tomatoes (Ahiakpa et al., 2020). In tea (*Camellia sinensis*), the *GIF* and *GRF* gene families could control the tea plant’s leaves by affecting the developmental process and hormonal stimuli responses (Wu et al., 2017). One gene, *CsGIF1,* showed a different expression pattern under abiotic stresses (Wang et al., 2017).

While *GIFs* have been studied in some plant species, this gene family also requires investigation in cotton. Cotton is an important industrial crop that provides natural fibre for textile industries and seed-soil for animal and human consumption. Recent advances in cotton genomics provide an opportunity to characterize the *GIF* gene family in *Gossypium*. Multiple high-quality genome data sets were available for several cotton species, such as *G. arboreum* (Li et al., 2014; Du et al., 2018), *G. hirsutum* (Li et al., 2015), *G. barbadense* (Wang et al., 2019), and *G. raimondii* (Paterson et al., 2012; Wang et al., 2012). In this study, we identified and characterized the *GIF* gene family in four cotton species by analyzing their gene structures, conserved motifs, cis-elements, and expression patterns. We further explored the role of *GIF* genes in response to waterlogging stress, and verified the function of *GhGIF4* in lateral root development by using a virus-induced gene silencing (VIGS) assay. The findings of this study will provide essential and valuable information for future studies on the functional characterization of the *GIF* gene family in cotton.

## 2 Materials and Methods

### 2.1 Identification of GRF1-Interacting Factor Homologs in *Gossypium*

Four *Gossypium* species (*G. arboreum*, *G. hirsutum*, *G.barbadense,* and *G. raimondii*) were used in this study. The genome and protein sequence files of those Gossypium species were downloaded from the cotton functional genome database (CottonFGD, https://cottonfgd.org/) (Zhu et al., 2017). The hidden Markov model (HMM) profile of the conserved GIF domain SSXT (PF05030) was obtained from PFAM (http://pfam.xfam.org). HMMER 3.0 (http://hmmer.org/) was used to search the four *Gossypium* genomes for *GIF* candidate genes. The redundant candidate genes were eliminated, and the remaining genes were further confirmed by SMART (http://smart.emblheidelberg.de/). Both of the protein physicochemical properties (including the number of amino acids, molecular weight (MW), and theoretical isoelectric point (pI) of GIF proteins) and chromosomal gene positions were obtained from CottonFGD (https://cottonfgd.org/).

### 2.2 Characterization and Sequence Analysis of Related *GIF* Genes

The chromosomal location of all identified *GIF* genes was visualized by TBtools (v1.098661) (Chen et al., 2020). The identified GIF protein sequences of cotton were aligned using the ClustarW program in MEGA5 software (Tamura et al., 2011). Afterward, the neighbor-joining (NJ) tree was constructed using MEGA5 by running 1,000 bootstrap replicates. The gene structure of *GIF* genes was analyzed by the gene structure display server (GSDS2.0, http://gsds.gao-lab.org/). The conserved structural motifs of *GIF* genes were analyzed with the MEME online software (http://meme-suite.org/tools/meme).

### 2.3 Prediction of Cis-Elements of *GIF* Genes

The 2000 bp upstream of the translation initiation site of each of *GIF* gene was extracted by TBtools (v1.098661) (Chen et al., 2020), and the *GIF* sequences were submitted to the PlantCARE online server (http://bioinformatics.psb.ugent.be/webtools/plantcare/html/) to predict their cis-elements.

### 2.4 Gene Expression Analysis of *GhGIFs*

TPM (Transcripts Per Kilobase of exon model per Million mapped reads) expression values of *GhGIFs* were obtained from CottonFGD’s online website (https://cottonfgd.org/). We analyzed the expression differences of *GhGIFs* under different tissues (root, stem, leaf, torus, pental, anther, bract, filament, pistil, and sepal) and different stress treatments (cold, drought, and salt treatments). The average TPM values for each sample were calculated and were visualized by Heatmap Illustrator HemI 1.0.3.7 (http://hemi.biocuckoo.org/). Genes with a TPM value fold change (treatment/control) > 2 were considered up-regulated genes, and fold change (treatment/control) < 0.5 were termed as down-regulated genes.

### 2.5 Plant Cultivation and Phenotypic Investigation

Two *G. hirsutum* accessions, Ji A-1-7 (33xi) and King, were selected to be cultured and investigated in this study. Cotton seeds were provided by the National Medium-term Gene Bank of Cotton in China. All the seeds were first soaked in water for 1 day and germinated in potting sand for 3 days. Then, the resulting seedlings were transferred into hydroponic containers with a half-strength of Hoagland nutrient solution. All the seedlings were maintained in controlled conditions at 26°C during the day and 23°C at night, with a 16 h light-to-8 h dark photoperiod. The root phenotype was observed. The primary root length and lateral root numbers were counted on the 8-day after sowing. The lateral root samples were collected and stored at −80°C for further qRT-PCR analysis.

### 2.6 Lateral Root Initiation Observation

We noticed that the lateral roots emerged at 96 h old. To better understand the lateral root initiation mechanism, we dissected the initiation region of the lateral root at 96 h after sowing. A 1.5 cm section of root samples were collected and mixed in FAA fixative for 24 h, then embedded in paraffin and stained with toluidine blue staining solution. The structures of cells for the lateral root initiation region were observed under a Leica upright microscope, Leica Dm6B.

### 2.7 CAT, MDA, and POD Measurements

The lateral root samples of 8 day-old seedlings were utilized to measure the Micro Catalase (CAT), Malondialdehyde (MDA), and Peroxidase (POD) content. Fresh cotton lateral root samples (0.1 g) were weighed into a 2 ml centrifuge tube with two steel balls and 1 ml of extract buffer, ground in an automatic sample cryo-grinder (Shanghai Jingxin Industrial Development Co., Ltd., China, Shanghai) at 4°C, centrifuged at 4°C × 12,000 rpm for 10 min, and the supernatant taken for further enzyme measurement. Then, the CAT, MDA, and POD activities were tested by their Kit (Micro Catalase (CAT) Assay Kit (Suzhou Grace Biotechnology Co., Ltd., China, Suzhou), Malondialdehyde (MDA) Assay Kit (Suzhou Grace Biotechnology Co., Ltd., China, Suzhou), and Peroxidase (POD) assay Kit (Suzhou Grace Biotechnology Co., Ltd., China, Suzhou)) according to the manual instructions.

### 2.8 qRT-PCR

The total RNA of cotton lateral roots and leaves were isolated by RNAprep Pure Plant kit (Tiangen, China) according to the manufacturer’s protocol. Approximately 1 μg of RNA was reversely synthesized into cDNA using the Prime Script TM RT reagent kit with g DNA Eraser kit (TaKaRa). For quantitative real-time PCR (qRT-PCR), we used Primer5 software to design primers for selected candidate genes (Supplementary Table S3), and those PCR products were checked by agarose gel electrophoresis. Later, the qRT-PCR was performed by the Roche LightCycler 480 II real-time PCR system using the PerfectStart Green qPCR SuperMix (Transgen Biotech Co.,Ltd.,). The *Histone3* gene was set as an internal control, and the relative expression of *GhGIFs* genes was calculated by the 2^−ΔΔCT^ method (Livak and Schmittgen, 2001).

### 2.9 Waterlogging Experiment

In order to clarify whether the *GhGIF* genes are affected by waterlogging, we conducted a waterlogging assay on the two representative cotton cultivars mentioned. The experiment used the sand culture method to grow seedlings. Seeds of Ji A-1-7 (33xi) and King were planted in a medium-size plastic box. Each box contained eight seeds, and four similar seedlings were selected and kept in each box on the ninth day. Both the two cotton cultivars contained 24 boxes, and half of them were used for waterlogging treatment. When cotton seedlings were grown to the period of the two-leaf and one-heart seedling stage, the treated boxes were added with water every day, so that the water surface was always about 1 cm higher than the sand surface. The control plants were watered according to the expected standard. Four weeks later, we observed the phenotype of all the plants and sampled leaves to detect the expression of three *GhGIF* genes (*GhGIF4*, *GhGIF5*, and *GhGIF8*) by qRT-PCR.

### 2.10 VIGS

A virus-induced gene silencing (VIGS) experiment was performed to verify whether *GhGIF* genes affect lateral root development in cotton. The VIGS primers are designed using https://crm.vazyme.com/cetool/multifragment.html (Supplementary Table S3). Briefly, a 300 bp fragment of *GhGIF4* was cloned into the *EcoRⅠ/BamHⅠ* sites of the pYL156 vector to construct the TRV: *GhGIF4* recombinant plasmid. The TRV: *GhGIF4* vector was transferred into *Agrobacterium tumefaciens* LBA4404. Seedlings of Ji A-1-7 (33xi) were cultured with a half-strength of Hoagland nutrient solution and were used as VIGS experimental plants. The A. tumefaciens strain LBA4404 was cultured overnight (14–18 h) in LB with rifampicin (30 μg/ml) and kanamycin (50 μg/ml) at 28°C with pYL156, pYL156: CLA1, pYL192, and transformed pYL156: GhGIF4. We adjusted the OD600 of the cell suspension to about 0.8 and let it continue to incubate in the dark for 3–4 h. Then, the *A. tumefaciens* carrying pYL156, pYL156: *CLA1,* and pYL156: *GhGIF4* was mixed with *A. tumefaciens* harboring pYL192 at 1:1, respectively. The *A. tumefaciens* mixed suspensions were infiltrated into the leaves of the Ji A-1-7 (33xi) on 5 days-old seedlings using a needleless syringe. Those plants were placed in a dark place for 24 h and then grew in the greenhouse at 26°C days and 23°C nights with a 16 hour-to-8 h dark cycle. About 2 weeks later, the *CLA1* plants were bleaching, and we observed the root morphology for all plants. The lateral root was collected for further analysis of qRT-PCR and enzyme activity (MDA, POD).

## 3 Results

### 3.1* GIF* Genes Identification and Chromosomal Locations in Four *Gossypium* Species

Combining the results of CottonFGD (https://cottonfgd.org/), HMMER3.1 (http://hmmer.org/), and SMART (http://smart.embl-heidelberg.de/), we identified a total of 12, 11, 6, and 6 full-length putative *GIF* genes in *G. hirsutum*, *G. barbadense*, *G. arboretum*, and *G. raimondii*, respectively. For convenience, the family *GIF* members of the four cotton species were renamed *GhGIF1* to *GhGIF12*; *GbGIF1* to *GbGIF11*; *GaGIF1* to *GaGIF6*; and *GrGIF1* to *GrGIF6*, respectively.

**FIGURE 1:**
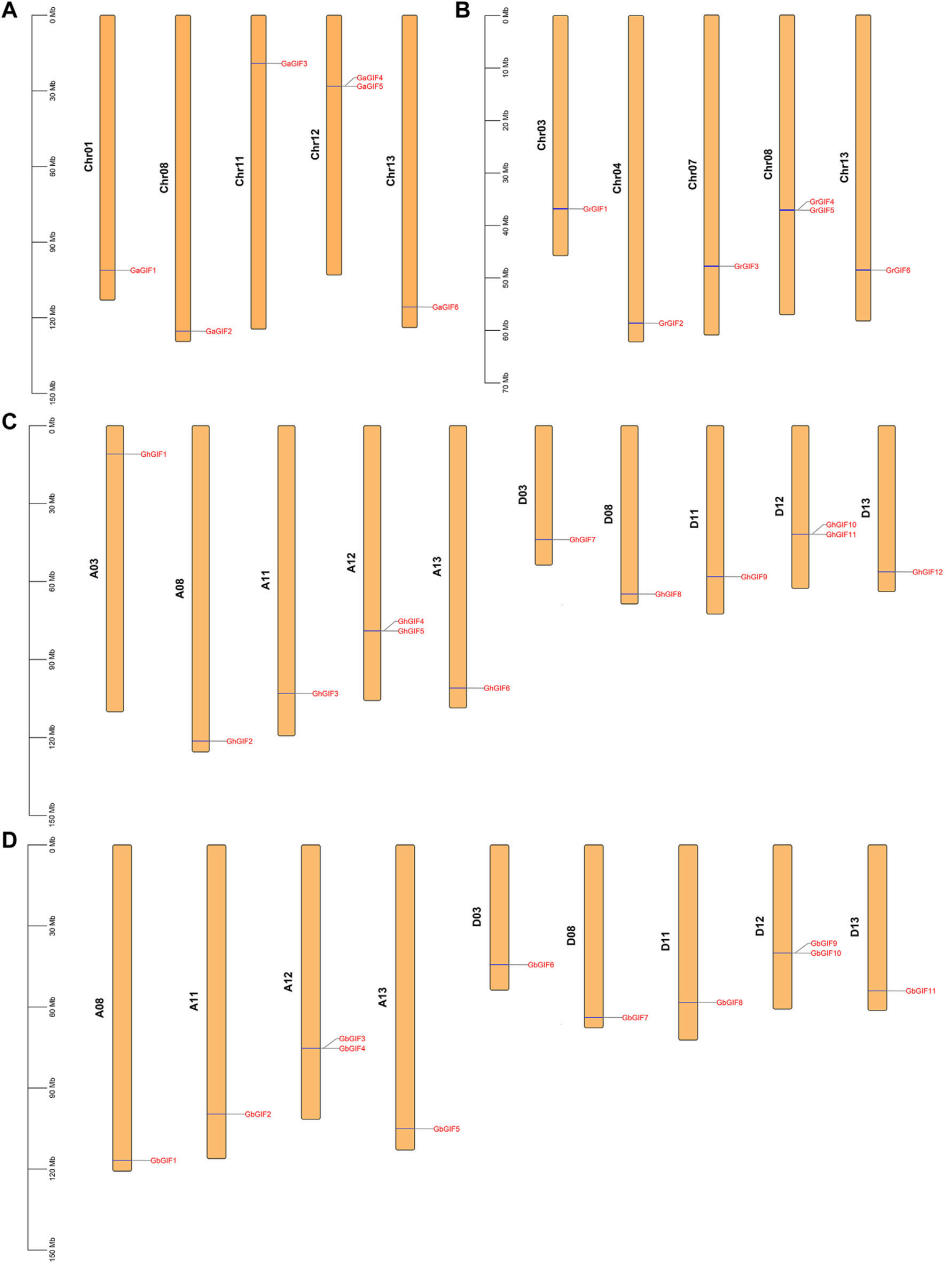
Chromosome distribution of *GIF* genes in four *Gossypium* species. **(A)** *G. arboreum* **(B)** *G. raimondii* **(C)** *G. hirsutum* **(D)** *G. barbadense.*

As shown in Figure 1, the *GIF* genes were unevenly distributed on different *Gossypium* chromosomes. In *G. arboretum* (Figure 1A), 2 GaGIF genes (*GaGIF4* and *GaGIF5*) were allocated on Chr12, while the other four *GaGIF* genes were distributed on Chr01, Chr08, Chr11, and Chr13, respectively. Similarly, two *GrGIF* genes (*GrGIF4* and *GrGIF5*) were observed on Chr08 in *G. raimondii*, and the other four *GrGIF* genes were observed on Chr03, Chr04, Chr07, and Chr13, respectively (Figure 1B). The *GIF* genes of *G. hirsutum* were distributed on ten chromosomes, five in subgroup At and five in subgroup Dt (Figure 1C). Furthermore, two adjacent *GIF* genes were distributed on the Chr12 chromosomes of the At and Dt subgroups both in *G. hirsutum* and *G. barbadense*. A total of 11 *GbGIF* genes were observed on nine chromosomes in *G. barbadense* (Figure 1D). Five of them were distributed among four At chromosomes (A08, A11, A12, A13), and six were distributed among five Dt chromosomes (D03, D08, D11, D12, D13).

Protein sequence analysis showed that all GIF proteins could encode 181–229 amino acids with a molecular weight (Mw) ranging from 19.255 to 23.948 kDa, and an isoelectric point (pI) ranging from 5.641 to 6.965 (Table 1, Supplementary Table S1). Subcellular mapping prediction indicated that all 35 *GIF* genes were located in the nucleus (Table 1).

**TABLE 1:** Characteristics of the putative cotton *GIF* genes.

Group	Gene ID	Gene name	Protein length (aa)	Molecular weight (kDa)	Isoelectric point	Subcellular location	Location
I	Ga08G2525	GaGIF2	181	19.255	6.384	Nucleus	125275931–125,278,631 (+)
I	Ga12G1762	GaGIF4	207	22.273	6.958	Nucleus	28,115,218–28,117,337 (+)
I	Ga12G1764	GaGIF5	213	22.587	6.506	Nucleus	28,142,143–28,145,063 (+)
I	GB_A08G2552	GbGIF1	181	19.285	6.384	Nucleus	116,777,991–116,780,695 (+)
I	GB_A12G1386	GbGIF3	213	22.557	6.506	Nucleus	75,268,736–75,271,651 (−)
I	GB_A12G1388	GbGIF4	207	22.273	6.958	Nucleus	75,293,981–75,296,101 (-)
I	GB_D08G2547	GbGIF7	182	19.291	6.717	Nucleus	63,889,525–63,892,226 (+)
I	GB_D12G1383	GbGIF9	213	22.557	6.506	Nucleus	40,012,500–40,015,438 (−)
I	GB_D12G1386	GbGIF10	207	22.334	6.965	Nucleus	40,033,628–40,035,709 (−)
I	Gh_A08G245300	GhGIF2	181	19.269	6.384	Nucleus	121,368,984–121,372,148 (+)
I	Gh_A12G132100	GhGIF4	213	22.557	6.506	Nucleus	78,978,869–78,982,370 (−)
I	Gh_A12G132300	GhGIF5	207	22.273	6.958	Nucleus	79,004,007–79,006,670 (−)
I	Gh_D08G236600	GhGIF8	182	19.276	6.717	Nucleus	64,790,318–64,793,402 (+)
I	Gh_D12G132400	GhGIF10	213	22.557	6.506	Nucleus	41,794,952–41,798,483 (-)
I	Gh_D12G132600	GhGIF11	217	23.409	6.718	Nucleus	41,821,393–41,824,062 (−)
I	Gorai.004G248600	GrGIF2	189	20.025	6.717	Nucleus	58,599,554–58,603,030 (+)
I	Gorai.008G129100	GrGIF4	213	22.539	6.506	Nucleus	37,154,435–37,158,202 (-)
I	Gorai.008G129300	GrGIF5	207	22.372	6.965	Nucleus	37,180,902–37,183,767 (−)
II	Ga01G2147	GaGIF1	188	20.686	6.511	Nucleus	101,144,538–101,146,150 (+)
II	Ga11G1054	GaGIF3	229	23.948	6.295	Nucleus	19,102,119–19,105,758 (−)
II	Ga13G2126	GaGIF6	211	22.615	6.628	Nucleus	1,15,718,453–1,15,719,686 (+)
II	GB_A11G2882	GbGIF2	229	23.915	6.293	Nucleus	99,617,921–99,621,536 (+)
II	GB_A13G2102	GbGIF5	211	22.572	6.387	Nucleus	105,088,627–105,089,859 (−)
II	GB_D03G1344	GbGIF6	186	20.628	5.641	Nucleus	44,329,950–44,331,566 (−)
II	GB_D11G2869	GbGIF8	228	23.933	6.413	Nucleus	58,251,001–58,254,587 (+)
II	GB_D13G2037	GbGIF11	211	22.587	5.837	Nucleus	53,978,796–53,980,027 (−)
II	Gh_A03G066000	GhGIF1	188	20.756	6.094	Nucleus	10,937,275–10,938,857 (+)
II	Gh_A11G282100	GhGIF3	229	23.916	6.506	Nucleus	103,037,699–103,041,896 (+)
II	Gh_A13G192000	GhGIF6	211	22.572	6.387	Nucleus	100,948,774–100,950,006 (−)
II	Gh_D03G131400	GhGIF7	189	20.966	5.641	Nucleus	43,817,417–43,819,406 (−)
II	Gh_D11G281800	GhGIF9	228	23.901	6.413	Nucleus	58,153,893–58,157,990 (+)
II	Gh_D13G195100	GhGIF12	211	22.587	5.837	Nucleus	56,284,974–56,286,209 (−)
II	Gorai.003G122900	GrGIF1	189	20.966	5.641	Nucleus	36,822,859–36,825,072 (−)
II	Gorai.007G279600	GrGIF3	228	23.933	6.413	Nucleus	47,854,934–47,859,486 (+)
II	Gorai.013G186300	GrGIF6	211	22.556	5.844	Nucleus	48,613,230–48,614,968 (+)

### 3.2* GIF* Gene Phylogenetic Tree, Conserved Motif, and Gene Structure Analysis

**FIGURE 2:**
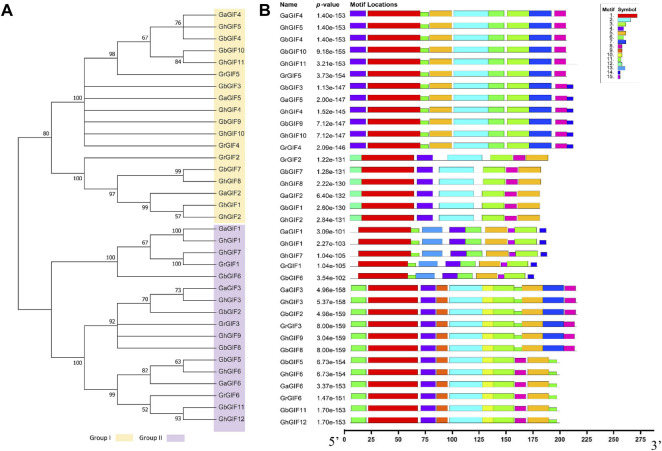
Phylogenetic and conserved motif analysis of *GIF* genes in *Gossypium*. **(A)** phylogenetic tree **(B)** The 35 *GIF* genes have 15 conserved motifs.

A phylogenetic tree was constructed using 35 GIF protein sequences from four *Gossypium* species to understand the evolutionary relationship between *GIF* genes better. Subsequent phylogenetic analysis revealed that *GIF* genes could be classified into two subfamilies (Group I and II). Group I contains 18 *GIF* genes, while Group II has 17 *GIF* genes (Figure 2A). Further, we identified fifteen conserved motifs among 35 cotton *GIF* genes using a MEME program (Figure 2B). The number of conserved motifs in *GIF* genes was different from 7 to 11. Motifs 1, 3, 4, and 5, were detected in all the *GIF* genes. All Group I members include Motif 2 and 8, and two-thirds of Group I members include Motif 6, 7, and 11. Moreover, all the Group II members include Motif 6 and 11, and 70.59% of Group II members include Motif 2, 9, and 10 (Figure 2B).

The structural gene variation of the *GIF* genes was then compared, yielding a comprehensive illustration of their evolutionary relationship. A variable structural pattern of exon-intron was observed in *GIF* family genes. As shown in Supplementary Figure S1, all the *GIF* genes have four CDS sequences, with three shorter and one longer. All of the *GIF* genes were identified with three introns. However, in Group I, the intron lengths of all *GIF* genes were similar; and in Group II, the intron lengths of *GaGIF3*, *GhGIF3*, *GbGIF2*, *GrGIF3*, *GhGIF8,* and *GhGIF9* are significantly longer than those of other *GIF* genes.

### 3.3 Cis-Element Analysis of *GIF* Promoters

**FIGURE 3:**
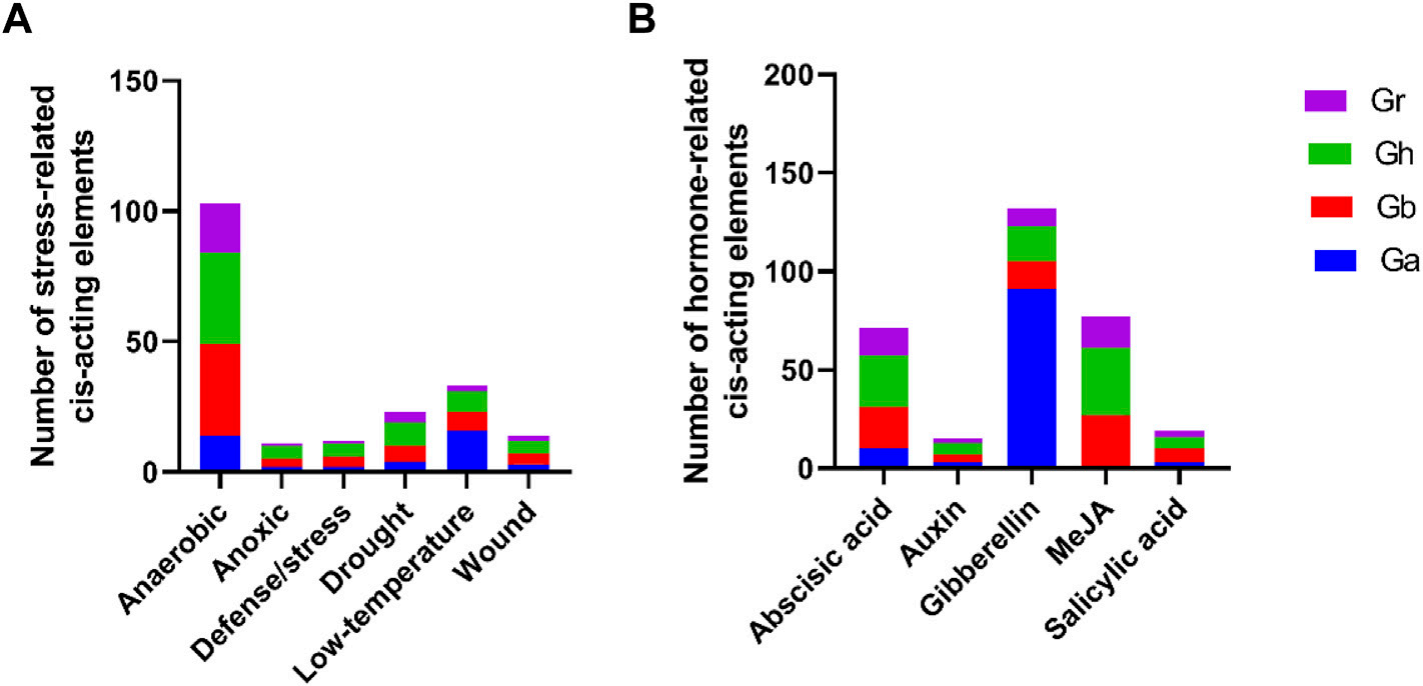
Cis-element analysis of *GIF* promoters in four *Gossypium* species. **(A)** analysis of the stress-related cis-acting elements **(B)** analysis of hormone-related cis-acting elements.

Cis-acting elements of promoters may play a crucial role in regulating of gene expression. In this study, we extracted the 2000 bp promoter sequence that was upstream of the start codon of each *GIF* gene and their cis-elements identification was due by using the PlantCare tool. We also screened out the stress and hormone-responsive elements for further analysis. As shown in Figure 3, the stress-related responsive mainly included anaerobic, anoxic, defense/stress, drought, low temperature and wound, and their cis-acting elements were ARE, GC-motif, TC-rich repeats, MBS, LTR, and WUN-motif respectively (Figure 3A, Supplementary Table S2). The majority were anaerobic responsive, and the representative *GIF* genes were *GhGIF4*, *GhGIF5*, *GhGIF3,* etc. The cis-elements of hormone-responsive mainly exhibited abscisic acid (ABRE), auxin (TGA-element), gibberellin (TATC-box, P-box, GARE-motif), MeJA (TGACG-motif, CGTCA-motif), and salicylic acid (TCA-element). The number of gibberellin-related cis-acting elements was the largest, and the *GIF* genes in *G.arboretum* occupied a larger ratio among them (Figure 3B; Supplementary Table S2).

### 3.4 Expression Analysis of *GhGIF* Genes

**FIGURE 4:**
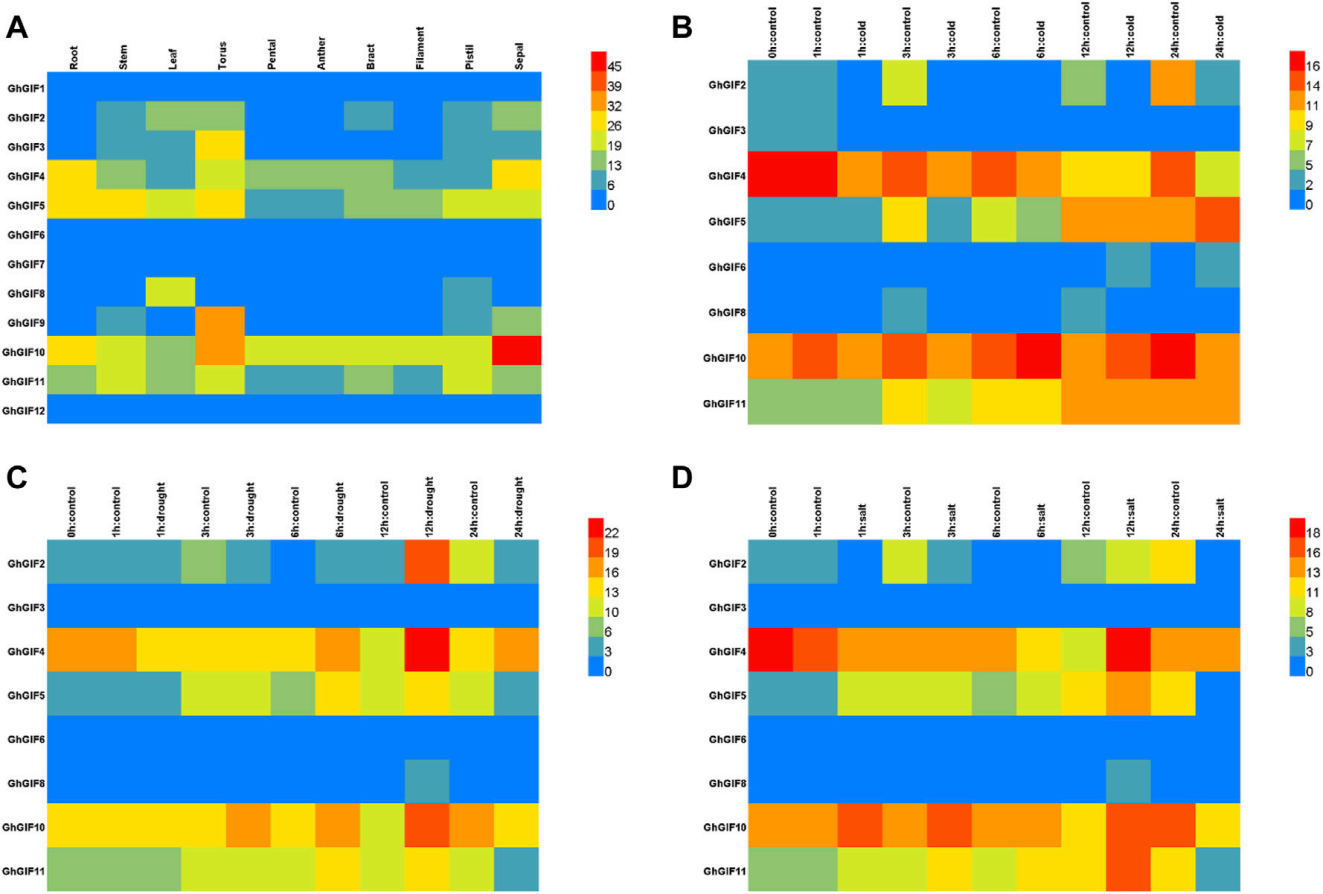
TPM expression of *GhGIF* genes in different organizations and different stresses. **(A)** TPM expression of *GhGIF* genes in different organizations in TM-1 **(B)** TPM expression of *GhGIF* genes under cold stress **(C)** TPM expression of *GhGIF* genes under drought stress **(D)** TPM expression of *GhGIF* genes under salt stress.

As shown in Figure 4, we compared the transcriptional patterns of *GhGIF* genes using multiple tissues and stresses of TM-1. Tissue expression pattern analysis showed that eight *GhGIF* genes (*GhGIF2*, *GhGIF3*, *GhGIF4*, *GhGIF5*, *GhGIF8*, *GhGIF9*, *GhGIF10*, and *GhGIF11*) exhibited different expression levels in various tissues, indicating that those *GIF* genes have complete biological functions in the growth and development of cotton (Figure 4A). Among the above *GIF* genes, four *GhGIFs* (*GhGIF4*, *GhGIF5*, *GhGIF10,* and *GhGIF11*) were highly expressed in root tissues, suggesting that these genes may play an essential role in root development. *GhGIF5* and *GhGIF8* are highly expressed in leaf tissues, which indicates that *GhGIF5* and *GhGIF8* may be involved in leaf development. At the same time, we also analyzed the expression level of *GhGIF* genes under cold, drought, and salt stress. Under cold stress treatment, the expression levels of *GhGIF2*, *GhGIF4*, and *GhGIF10* genes were down-regulated, and the longer the cold treatment, the greater the degree of down-regulation. In contrast, the expression of *GhGIF5* would gradually increase after cold treatment (Figure 4B). Under drought stress, the expression of *GhGIF4* would gradually increase after 6 h of treatment and reach its maximum at 12 h. Similarly, the expression of *GhGIF10* was up-regulated during the three periods of drought (3, 6, and 12 h), and reached its maximum at 12h, but we detected a down-regulation of this gene at 24 h. In addition, three genes of *GhGIF2*, *GhGIF5,* and *GhGIF11* also exhibited different expression levels in drought stress, indicating that these genes may be involved in drought stress (Figure 4C). Five genes of *GhGIF2*, *GhGIF4*, *GhGIF5*, *GhGIF10* and *GhGIF11,* exhibited a varied expression in different salt treatments, which indicated that those genes might play an important role in salt stress (Figure 4D).

### 3.5 Evaluation of the Lateral Root Phenotype in Ji A-1-7 (33xi) and King

**FIGURE 5:**
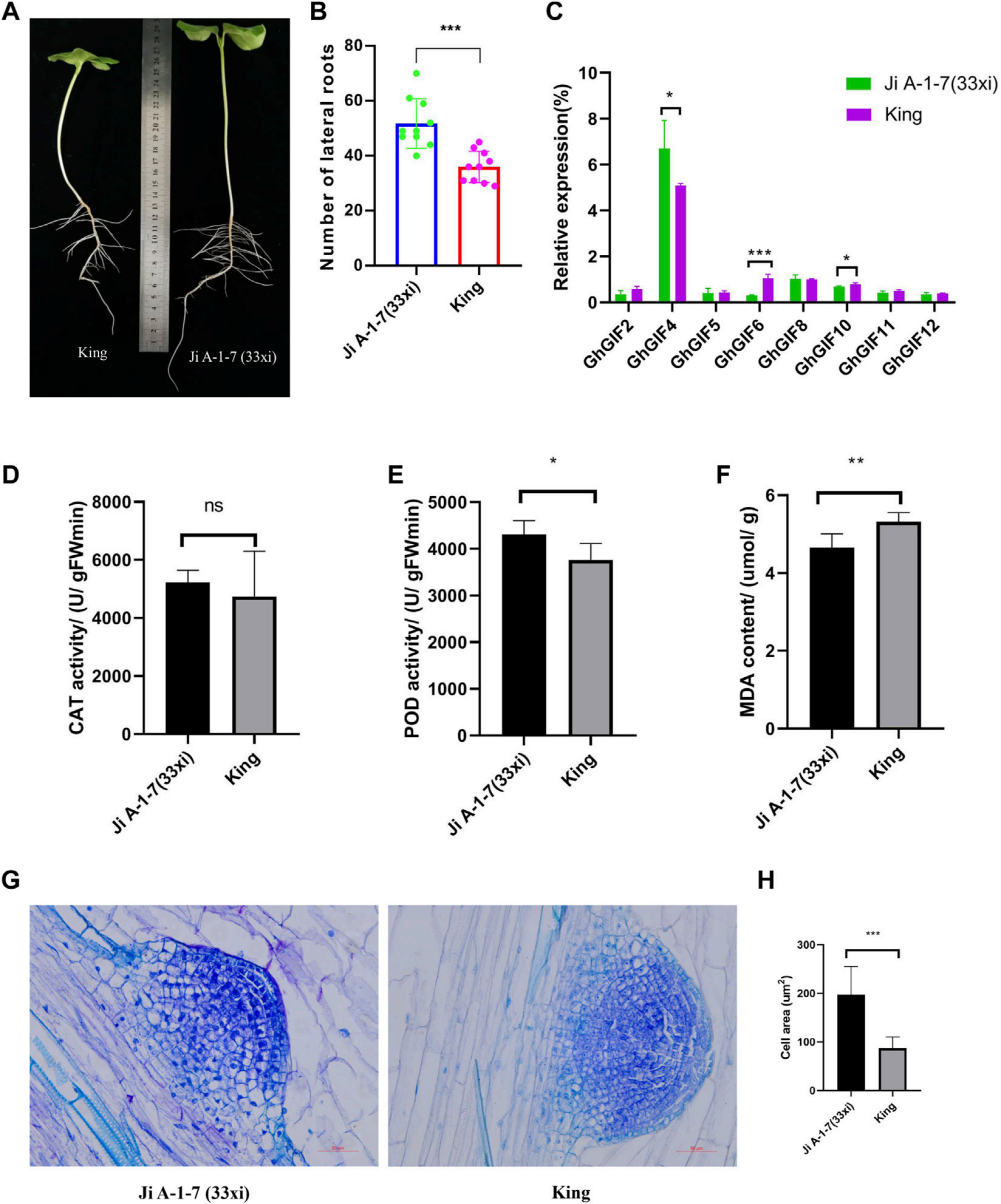
Comparison of lateral roots between Ji A-1-7 (33xi) and King. **(A)** Phenotype of Ji A-1-7 (33xi) and King on the 8-days after sowing **(B)** Comparison of lateral root numbers **(C)** Relative expression of eight *GhGIF* genes in lateral roots **(D)** CAT activity of lateral roots **(E)** POD activity of lateral roots **(F)** MDA content of lateral roots **(G)** microstructure of the lateral root emergence part in Ji A-1-7 (33xi) and King **(H)** Comparison of cell area between Ji A-1-7 (33xi) and King.

Ji A-1-7 (33xi) showed significantly larger lateral roots than King (Figures 5A,B). Further qRT-PCR expression results showed that three genes of *GhGIF4*, *GhGIF6,* and *GhGIF10,* expressed significantly different expression levels in lateral roots between Ji A-1-7 (33xi) and King with the highest expression value recorded for *GhGIF4* (Figure 5C)*.* The POD, CAT, and MDA results showed no difference in the CAT activity between the above two accessions. However, a significant difference was detected for POD activity and MDA content. The lateral root’s POD activity is higher in Ji A-1-7 (33xi), but King’s MDA content is higher (Figures 5D–F). The paraffin sectioning assay indicated that Ji A-1-7 (33xi) had more giant cells than King with the average cell area in Ji A-1-7 (33xi) being significantly higher than King on the lateral root emergence part (Figures 5G,H).

### 3.6 Waterlogging Stress Experiment

**FIGURE 6:**
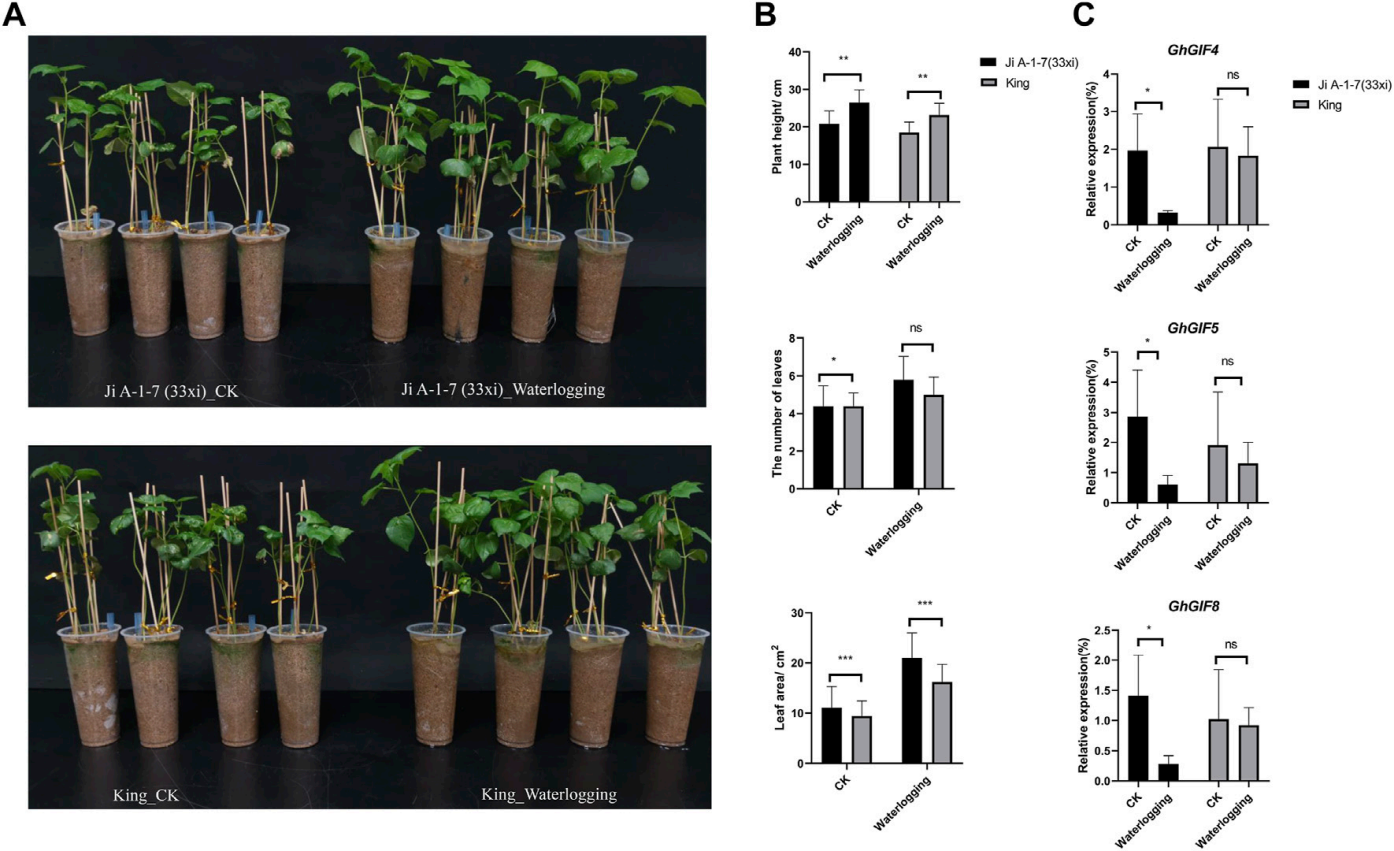
Response of *GhGIF* genes to waterlogging. **(A)** The phenotype of Ji A-1-7 (33xi) and King under waterlogging stress **(B)** Count of plant height, lateral root numbers, and the leaf areas **(C)** Relative expression of *GhGIF4*, *GhGIF5*, and *GhGIF8* in leaves of waterlogging and its control (CK) seedlings.

Surprisingly, we found that the waterlogged plants grew more prosperous than the control plants. The plant height, leaf numbers and areas (last second leave) were significantly higher than those of control plants (Figures 6A,B). Unfortunately, we failed to count the lateral root numbers due to their easy breakage when washing their surface sand soil. *GhGIF4*, *GhGIF5*, and *GhGIF8* expression levels in the leaf of Ji A-1-7 (33xi) showed significantly higher in the waterlogging plants than in the control (Figure 6C). Expression levels of those genes in King also showed a similar pattern but did not reach a significant level (Figure 6C).

### 3.7 *GhGIF4* Silencing in Ji A-1-7 (33xi)

**FIGURE 7:**
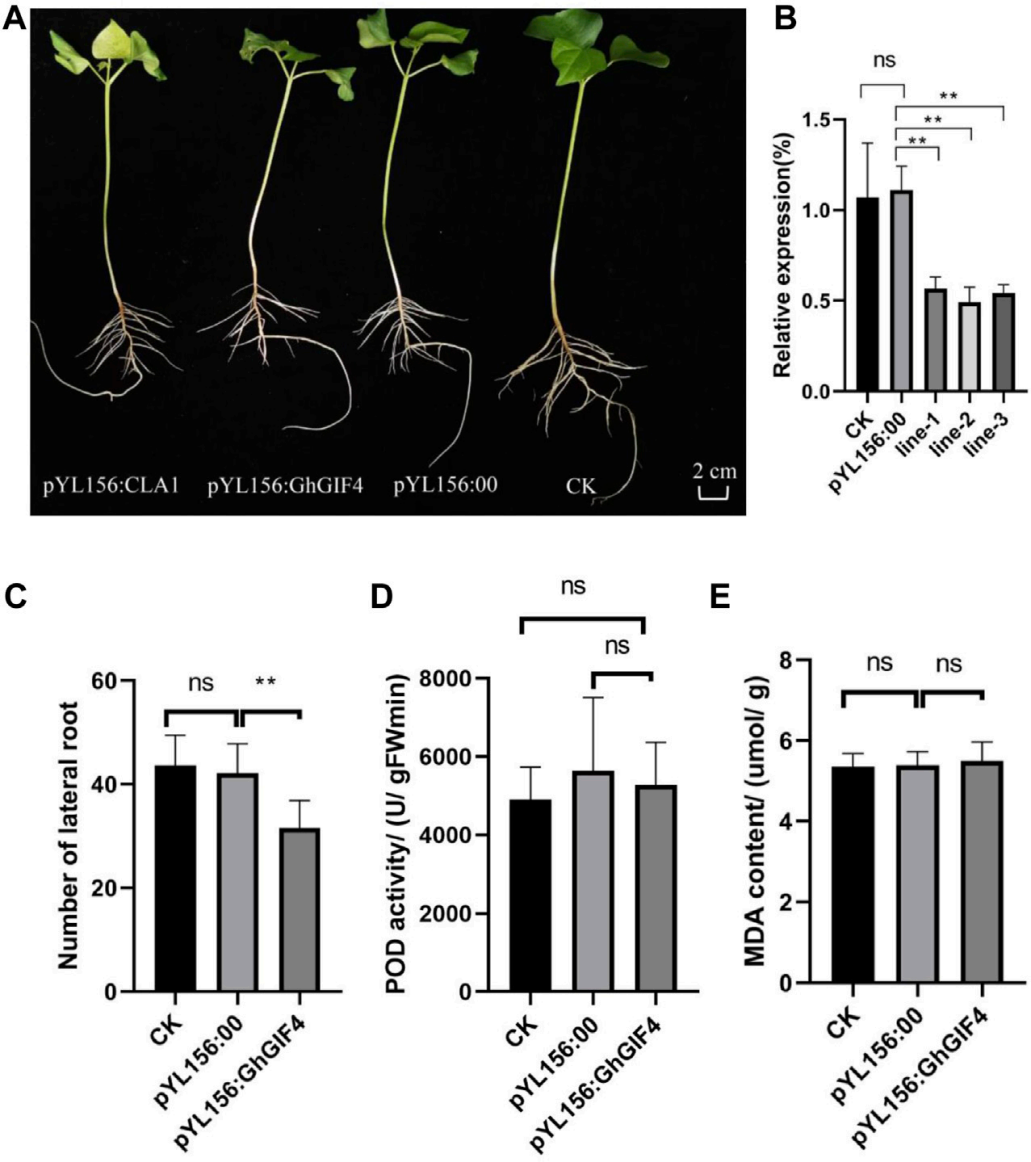
Virus-induced Gene Silencing (VIGS) of *GhGIF4* in cotton. **(A)** The phenotype of the gene silencing plants **(B)** Relative expression of *GhGIF4* in silenced and its control plants **(C)** The number of lateral roots **(D)** POD activity **(E)** MDA content. Note, all values represented the mean ± s.d. (n≥3 replicates), ns represent not significant, ∗ represent significance at *p* < 0.05 level.

There were three *GIF* genes with high expression levels related to lateral development, while three *GIF* genes varied in response to waterlogging stress. Among the *GIF* genes, the common key gene is *GhGIF4*, resulting in that we further evaluate the function of this gene on lateral root development in cotton by VIGS. We observed the silenced seedlings and their controls when the positive control plants that injected the recombinant vector pYL156:*CLA1* showed an albino phenotype (Figure 7A). The expression of *GhGIF4* in silenced plants was significantly lower than the negative (CK) and positive (pYL156:00) controls. Moreover, the silencing efficiency of VIGS is about 40–60% (Figure 7B). The number of lateral roots in silenced plants was also significantly lower than in their controls (Figure 7C). Later, we also compared the lateral roots’ POD activity and MDA content between the silenced plants and their controls. A similar result was observed for the POD activity and MDA content (Figures 7D,E). Therefore, we speculate that *GhGIF4* may positively regulate cotton lateral root numbers but not be influenced by the POD activity and MDA content.

## 4 Discussion

Recently, several high-quality genomes of *Gossypium* including *G. arboreum*, *G. raimondii*, *G. hirsutum* and *G. barbadense* have been released, which would provide valuable information for us to study the *GIF* family in cotton. Previous studies mainly reported the *GIF* genes were involved in plant growth and development, such as controlling the leaf, seed, and root meristem homeostasis in *Arabidopsis* (Rodriguez et al., 2010; Debernardi et al., 2014; Liang et al., 2014; Ercoli et al., 2018); modulating the tissue and organ size in rice (Song et al., 2011; Duan et al., 2015; Li et al., 2016); and regulating the shoot architecture and meristem determinacy in maize (Zhang et al., 2018). Here, we identified a total of 35 *GIF* genes and analyzed their structures and phylogenetic relationships in four *Gossypium* species. We further performed preliminary studies to verify the response of *GIF* genes to adversity stress and the effect of *GhGIF4* on cotton lateral root development. Our results have been provided new insights into GIF proteins for cotton.

Previous reports well described that *G. arboretum* could be seen as the ancestor of *G. hirsutum* by providing an A subgenome (Kebede et al., 2007). Intriguingly, in this study, we found that the number of *GIF* genes identified in *G. hirsutum* was twice as much as that in *G. arboreum*. Their distributions were consistent with the cotton allotetraploid evolutionary process (Flagel and Wendel, 2009). So, we speculated that the *GIF* genes were multiplied by the polyploidy event of subfamily At and Dt during the formation of *G. hirsutum*. Furthermore, this speculation was also suited to the *GIF* genes identified in *G. raimondii* and *G. barbadense*. However, only 11 *GbGIF* genes were identified in *G.barbadense,* with five on four At chromosomes (A08, A11, A12, A13) and six on five Dt chromosomes (D03, D08, D11, D12, D13). It seems that there should be one *GbGIF* gene on the A03 chromosome. We have not identified any *GIF* genes on the A03 in *G. barbadense,* and the reason may be due to the occurrence of gene loss events. Based on the phylogenetic analysis, those 35 GIF protein sequences identified in the four cotton species can be classified into two groups: Group I and Group II. We further analyzed the conserved motif and gene structures of those GIF proteins, and the result showed that both the conserved motifs and their gene structures are relatively conservative. Four motifs of motif_1, motif_3, motif_4, and motif_5 were all detected in all the 35 GIF proteins.

Moreover, all the *GIF* genes in *Gossypium* have four CDS sequences and three introns. However, the intron length was different between Groups I and II. The different structures of those *GIF* genes between Group I and Group II implied that the varied functions of *GIF* genes might be closely related to evolution.

Currently, upland cotton is the largest natural fiber crop globally. However, during its vegetative and reproductive periods, its yield and fiber quality are adversely affected by abiotic stresses such as high temperatures, cold, drought, waterlogging, and salt stress. Recently, the *GIF* genes were also involved in abiotic stresses. For example, Wang et al. (2017) cloned a *CsGIF1* gene in tea (*Camellia sinensis*) and found the *CsGIF1* had different expression patterns under different abiotic stresses (Wang et al., 2017). In this study, our results mainly found that *GhGIF2*, *GhGIF4* and *GhGIF10* were always down-regulated in the cold stress treatment, and those genes may be closely regulated in response to cold stress. *GhGIF4* and *GhGIF10* may also respond to drought stress due to their specific expression patterns. Five genes, *GhGIF2*, *GhGIF4*, *GhGIF5*, *GhGIF10,* and *GhGIF11, may* respond to salt stress. In the past 2 years, floods have frequently occurred in Henan, China, which has caused severe yield and quality losses to the local cotton. However, experiments with waterlogging are rare in cotton. We tried a mild waterlogging treatment on two representative cotton accessions in this study. Surprisingly, we found cotton seedlings treated with waterlogging grew more vigorously than control plants, with higher plant height and more vigorous leaves. Of course, in the actual field, experienced growers know that vigorous leaves and higher plant heights are not conducive to the final yield of cotton. From this perspective, our results are reasonable, and we speculated that severe floods would cause the hypoxic death of seedlings and further severely affect the yield of cotton. We also detected the expression levels of *GhGIF4*, *GhGIF5*, and *GhGIF8*, and the result demonstrated that those genes were down-regulated in the waterlogged seedlings, especially for Ji A-1-7 (xi). Therefore, we hypothesized that the *GIF* genes of *GhGIF4*, *GhGIF5*, and *GhGIF8* may respond to waterlogging in upland cotton.

A recent study showed that *AN3/GIFs* might be involved in different pathways that control the quiescent centre (QC) organization and the meristem size in *Arabidopsis* (Ercoli et al., 2018). In this study, we found that the gene for *GhGIF4* may affect the lateral root numbers in upland cotton. The relative *GIF* gene expressions, enzyme activity, and microstructure of the emergence part of the lateral roots of Ji A-1-7 (33xi) and King were also compared. Among the eight selected *GhGIF* genes, *GhGIF4* was expressed the highest and showed a significant difference between the two representative accessions. The lateral root’s POD activity in Ji A-1-7 (33xi) was higher, while the MDA content in King was higher. Moreover, the microstructure assay showed that the lateral root of Ji A-1-7 (33xi) had more giant cells than King on the lateral root emergence part. Furthermore, a further VIGS assay for *GhGIF4* showed smaller lateral root numbers than control, which emphasized the vital role of *GhGIF4* in lateral root development in upland cotton.

## 5 Conclusion

In this study, we identified 35 *GIF* genes in four cotton species. We analyzed their gene structures, phylogenetic relationships, conserved domains, cis-elements, and expression patterns in various organizations and different stresses. The qRT-PCR of *GIF* genes in lateral roots showed that the *GhGIF4* expressed higher and significantly differed between Ji A-1-7 (33xi) and King, which had larger and smaller later root numbers, respectively. The enzyme activity and microstructure of the emergence part of the lateral roots in those two cotton accessions were also compared. The result showed that Ji A-1-7 (33xi) had a higher POD activity and lowered MDA content and more giant cells of the lateral root phenotype than King. A mild waterlogging assay showed that the waterlogging-seedlings grew a more vigorous phenotype than control, and three selected *GIF* genes were all down-regulated. A VIGS assay of *GhGIF4* further verified the vital role of *GhGIF4* in lateral root development in the cotton. Together, our findings provide a foundation for further functional studies of the *GIF* genes in cotton plant growth, lateral root development, and stress tolerance.

## Data Availability

The original contributions presented in the study are included in the article/Supplementary Material, further inquiries can be directed to the corresponding author.
