# Supplementary figures and images for: Identification and Characterization of the Growth-Regulating Factors-Interacting Factors in Cotton
Source: Front Genet. 2022 Mar 14;13:851343. doi: 10.3389/fgene.2022.851343 (PMC8964071; doi:10.3389/fgene.2022.851343)

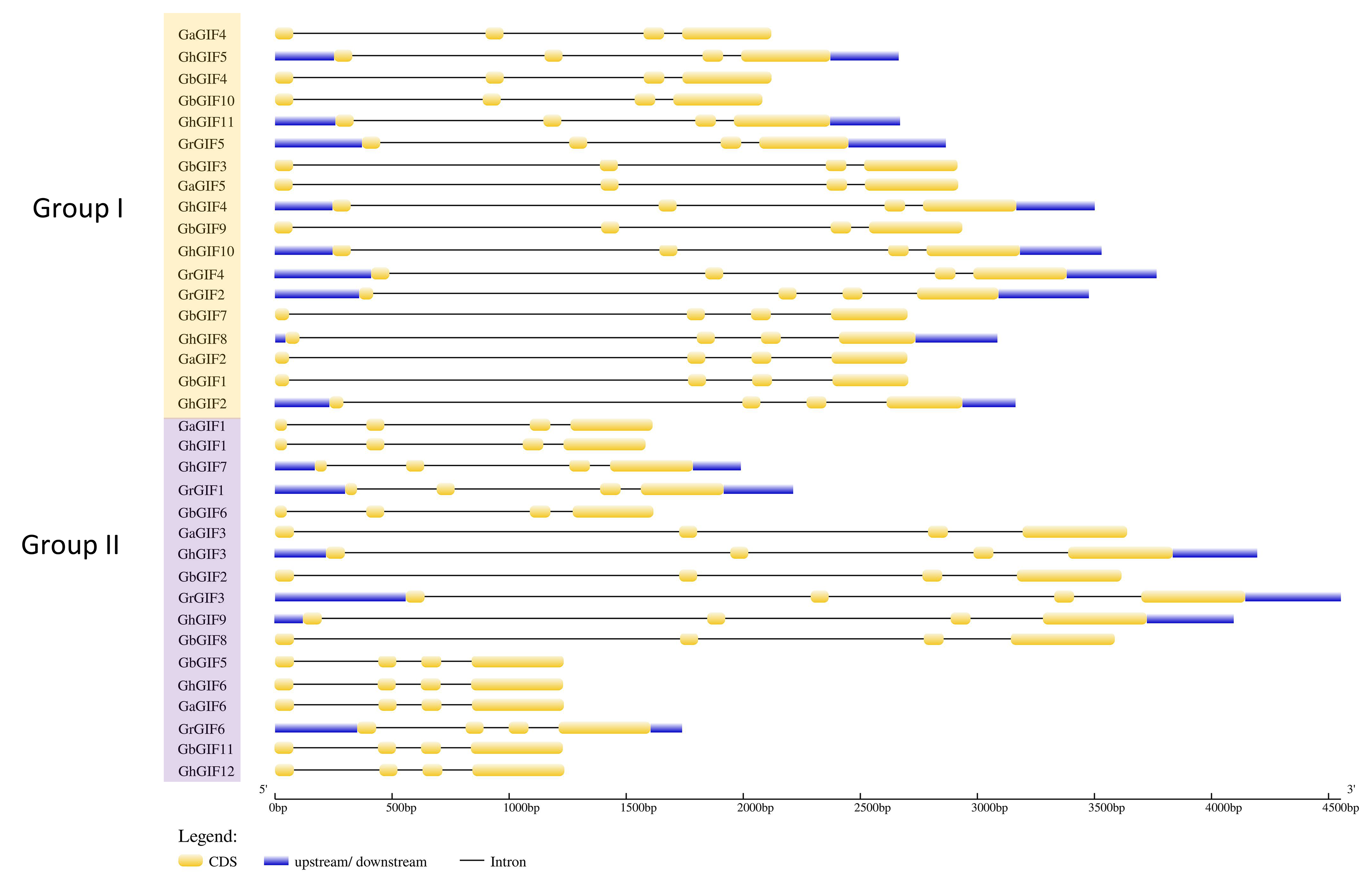

Supplement: Supplementary file 1 [file Image1.TIF]
